# Cullin-RING ubiquitin ligases: global regulation and activation cycles

**DOI:** 10.1186/1747-1028-3-7

**Published:** 2008-02-18

**Authors:** Dimple R Bosu, Edward T Kipreos

**Affiliations:** 1 Department of Cellular Biology, University of Georgia, 724 Biological Sciences Bldg., Athens, GA 30602-2607, USA

## Abstract

Cullin-RING ubiquitin ligases (CRLs) comprise the largest known category of ubiquitin ligases. CRLs regulate an extensive number of dynamic cellular processes, including multiple aspects of the cell cycle, transcription, signal transduction, and development. CRLs are multisubunit complexes composed of a cullin, RING H2 finger protein, a variable substrate-recognition subunit (SRS), and for most CRLs, an adaptor that links the SRS to the complex. Eukaryotic species contain multiple cullins, with five major types in metazoa. Each cullin forms a distinct class of CRL complex, with distinct adaptors and/or substrate-recognition subunits. Despite this diversity, each of the classes of CRL complexes is subject to similar regulatory mechanisms. This review focuses on the global regulation of CRL complexes, encompassing: neddylation, deneddylation by the COP9 Signalosome (CSN), inhibitory binding by CAND1, and the dimerization of CRL complexes. We also address the role of cycles of activation and inactivation in regulating CRL activity and switching between substrate-recognition subunits.

## Introduction

Protein degradation is critical for the regulation of a large number of diverse cellular processes. The majority of protein degradation in cells occurs via the ubiquitin-mediated proteolytic pathway [1,2]. Ubiquitin is an evolutionarily conserved 76 amino acid polypeptide that is covalently attached to target proteins by the concerted actions of three classes of enzymes [3,4]. A ubiquitin-activating enzyme (E1) utilizes one ATP to bind ubiquitin via a thiolester linkage. The activated ubiquitin is then transferred to a ubiquitin-conjugating enzyme (E2). E2s interact with ubiquitin-protein ligases (E3s), which also bind the substrate. The E3 brings the E2 and the substrate together. The E2 can then either directly conjugate ubiquitin to the substrate or, in the case of HECT-domain E3s, transfer the ubiquitin as a high-energy thiol intermediate to the E3, which then transfers it to the substrate. The attachment of a single ubiquitin to a substrate can alter protein function or localization [5]. The tandem attachment of multiple ubiquitin to form a polyubiquitin chain can also alter function or localization, or mark the substrate for degradation by the 26S proteasome, depending on the type of linkage within the polyubiquitin chain [6].

Ubiquitin ligases provide the substrate specificity for ubiquitination (ubiquitylation) reactions. The largest known class of ubiquitin ligases are cullin-RING ubiquitin ligases (CRLs) [7]. CRLs regulate diverse cellular processes, including multiple aspects of the cell cycle, transcription, signal transduction, and development [7]. CRLs are multisubunit complexes that include a cullin, a RING H2 finger protein, a substrate-recognition subunit (SRS), and with the exception of CUL3-based CRLs, an adaptor subunit that links the SRS to the complex. There are five major categories of cullins in metazoa (CUL1 through CUL5) [8,9], and an additional, potentially vertebrate-specific class containing CUL7 and PARC (Parkin-like cytoplasmic protein) [10]. CRLs are activated by the covalent attachment of the ubiquitin-like protein Nedd8 to the cullin, and are inhibited by binding to the CAND1 inhibitor [7]. Recently, it has become apparent that many CRLs function as dimers, which is another potential source of regulation. This review describes the global regulatory mechanisms that govern CRL activity, and highlights the current gaps in our understanding.

### The structure of CRL complexes

**Figure 1:**
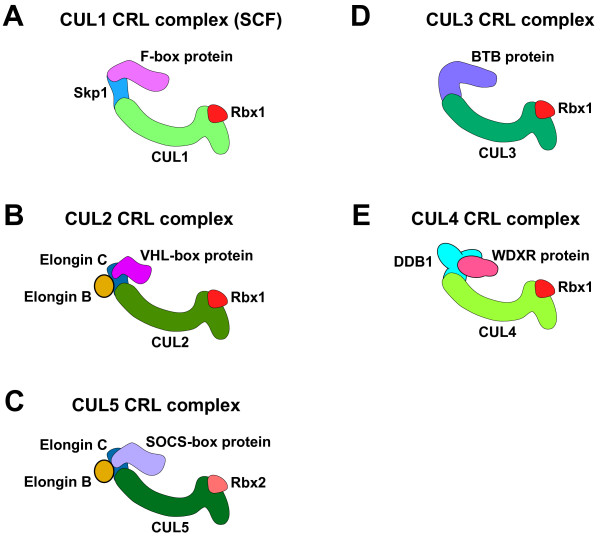
**Structures of multisubunit CRL complexes**. Diagrams of the CUL1 (A), CUL2 (B), CUL5 (C), CUL3 (D), and CUL4 (E) CRL complexes. Proteins in the complexes are labeled. The structures are described in the text.

The most intensively studied cullin is metazoan CUL1 and its budding yeast ortholog Cdc53. CUL1 and Cdc53-based CRLs are called SCF complexes, and contain four subunits: Skp1; CUL1 (Cdc53); an F-box protein; and the RING H2 finger protein Rbx1/Roc1/Hrt1 [7]. The crystal structure of the SCF complex reveals that the cullin acts as a rigid backbone for the assembly of the complex [11,12] (Fig. 1A). The CUL1 C-terminus binds Rbx1 and the N-terminus binds the adaptor Skp1. Rbx1 facilitates the recruitment of the E2 to the complex [13]. The adaptor Skp1 binds the SRS, which is an F-box protein that links to Skp1 through the F-box motif. The F-box protein binds and positions the substrate for ubiquitination by the E2. The combination of distinct F-box proteins with the core components creates unique SCF complexes that bind distinct sets of substrates. Metazoan genomes contain a relatively large number of genes encoding F-box proteins, e.g., humans have ~70 F-box proteins, while *C. elegans *has over 300 [14,15]. Many uncharacterized yeast and mammalian F-box proteins are capable of forming SCF complexes *in vitro*, suggesting the existence of a large number of SCF complexes [16,17]. F-box proteins generally bind to phosphorylated residues on substrates, and therefore, substrate degradation by SCF complexes is regulated by phosphorylation [7].

CUL2-based CRL complexes have a structure similar to that of the SCF complex (Fig. 1B). Rbx1 similarly binds to the C-terminus of CUL2, and the adaptor Elongin C binds to the N-terminus [18,19]. Elongin C is a Skp1-related protein that binds the complex as a heterodimer with the ubiquitin-related protein Elongin B [20]. SRSs bind to Elongin C through a VHL-box protein motif in the SRS [21].

CUL5 is the closest paralog to CUL2 [9], and CUL5 CRLs have a structure similar to that of CUL2 CRLs [21,22] (Fig. 1C). Both CUL-2 and CUL-5 CRLs employ Elongin C as the adaptor protein. Despite containing the same adaptor protein, CUL2 and CUL5 complexes bind different classes of SRSs. CUL5 complex SRSs utilize the SOCS-box motif to bind to Elongin C. The SOCS-box motif is similar to the VHL-box motif of CUL2 complex SRSs. Both motifs have an N-terminal subdomain (the BC-box) that binds Elongin C. However, the C-terminal regions of the motifs are distinct: the SOCS-box has a CUL5-box subdomain; and the VHL-box has a CUL2-box subdomain. These C-terminal subdomains are proposed to bind to the relevant cullin and thereby provide specificity [21,23]. CUL5 CRL complexes also utilize the RING H2 finger protein Rbx2/Roc2 rather than the related Rbx1, which is present in the other classes of CRLs [24].

CUL3 CRL complexes contain Rbx1, but differ from other CRL classes in that there is no adaptor protein (Fig. 1D). Instead, the SRS binds directly to the N-terminus of CUL3 using a BTB/POZ domain [25-28]. There are hundreds of BTB proteins in metazoan species, suggesting large numbers of distinct CUL3 complexes [7].

CUL4 CRL complexes contain Rbx1 and the adaptor protein DDB1 [29,30] (Fig. 1E). DDB1 binds to SRSs that contain WD-repeats of a subclass called 'WDXR' or 'DXR', which mediate interaction with DDB1 [31-34]. In at least one case, DDB1 has been reported to bind a substrate directly, providing the possibility that DDB1 can function as both an adaptor and an SRS [35].

The nomenclature for CRLs is only well established for CUL1-based SCF complexes. The naming of other CRL complexes is not settled, with various competing acronyms. For this review, we will refer to non-SCF CRL complexes by using the acronym "CRL" for cullin RING ubiquitin ligase followed by the number of the cullin, and a superscript to denote the SRS. Therefore, a CUL3 CRL complex with the SRS Keap1 will be referred to as CRL3^Keap1^.

### Dimerization of CRLs

**Figure 2:**
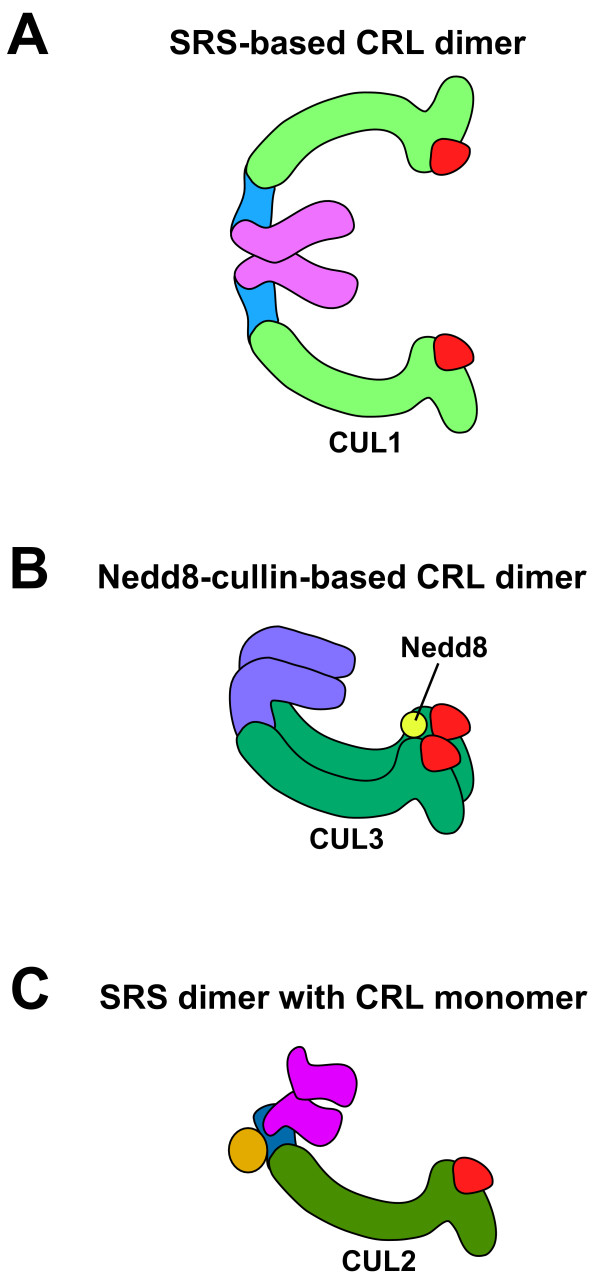
**Proposed models for dimerization of CRL complexes**. (A) Diagram of an SRS-mediated dimeric SCF complex. Dimerization is mediated by interactions between the SRSs in each CRL. This structure has been experimentally confirmed [38]. (B) Diagram of a Nedd8-cullin-based dimeric CRL3 complex. Dimerization is mediated by interaction between Nedd8, which is covalently linked to one CUL3 protein, and the WH-B domain of an unneddylated CUL3 [37]. The overall structure of the Nedd8-cullin-based dimer has not been determined. The dimer is drawn in a head-to-head conformation to accommodate the binding of a dimeric SRS to the two CUL3 N-termini (as many CRL3 SRSs are constitutively dimeric *in vivo*). (C) Diagram of a monomeric CRL2 complex binding a dimeric SRS. The existence of such a structure has not yet been directly confirmed by experiments (see text). Proteins are labeled as in Fig. 1.

Recently it has become apparent that a number of CRL complexes function as dimers. CUL1, CUL3 and CUL4-based CRL complexes have been observed to form dimers or multimers *in vivo *[36-38]. In contrast, CUL2 and CUL5 CRL complexes have only been observed as monomers [36]. There are two potential mechanisms of dimerization: SRS-mediated dimerization (which has been demonstrated for SCF complexes); and a Nedd8-cullin linkage (which has been demonstrated for CUL3 CRL complexes). SRS-mediated dimerization relies on binding between SRS proteins to link together two CRL complexes. Multiple F-box proteins have been observed to form dimers *in vivo*, including Fbw7, Pop1 & Pop2, Cdc4, Met30, Skp2, and βTrcp1 & βTrcp2 [39-45]. Dimerization of F-box proteins is initiated through a conserved D-domain located immediately N-terminal of the F-box motif [38,42,44]. Analysis of SCF^Cdc4 ^complexes by small angle X-ray scatter analysis indicates that the two substrate-binding sites of the SRSs and the two E2-binding sites form a coplanar surface in a suprafacial orientation [38] (Fig. 2A).

**Figure 3:**
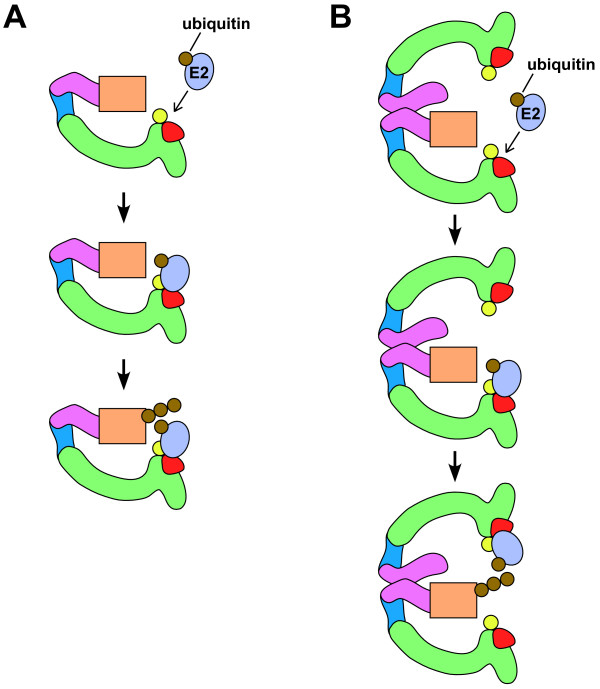
**Poly-ubiquitination reactions by monomeric and dimeric SCF complexes**. Diagram of poly-ubiquitin conjugation to a substrate (rectangle) by monomeric (A) and dimeric (B) SCF complexes. Top panels, E2 with activated ubiquitin prior to binding. Middle panels, E2 with activated ubiquitin loaded onto E3 but prior to transfer of ubiquitin to substrate. Bottom panels, the substrate has a three-ubiquitin chain and a new E2 with activated ubiquitin has docked. Note how the ability of E2s to load onto both sites of the dimeric SCF complex facilitates the addition of ubiquitin onto the growing polyubiquitin chain. In the diagram, the addition of the first ubiquitin is more sterically favorable from the E2 docking site that is closer to the substrate, while additions to the elongated polyubiquitin chain are more favorable from the more distant E2 docking site. Proteins are labeled as in Fig. 1.

The bivalent geometry of the dimeric SCF structure provides different distances between a substrate-binding site and the two E2 docking sites [38] (Fig. 3B). These distinct catalytic site-to-substrate distances can allow an SCF complex to target different-sized substrates and accommodate changes in the length of the elongating polyubiquitin chain [38] (Fig. 3). For the SCF^Cdc4 ^complex, dimerization does not affect its affinity for the substrate Sic1, but is required for optimal ubiquitin chain initiation and elongation [38,44]. The *in vitro *ubiquitination of three of four tested SCF^Cdc4 ^substrates is more efficiently ubiquitinated by dimeric SCF^Cdc4 ^[38]. Similarly, dimeric mammalian SCF^Fbw7/hCdc4 ^can more efficiently ubiquitinate its substrate cyclin E than can monomeric SCF^Fbw7/hCdc4 ^[44]. Dimerization also provides the potential for the two SRSs in the complex to work together to bind one substrate so that it is optimally tethered for ubiquitination, as has been proposed for the dimeric CRL3^Keap1 ^complex binding to its substrate Nrf2 [46].

SRSs can bind to SCF complexes as both homodimers and heterodimers. The F-box proteins βTrCP1 and βTrCP2 form both homo and heterodimeric complexes, but only the homodimeric forms of each can ubiquitinate the substrate Iκ Bα [39]. In contrast, the fission yeast F-box proteins Pop1 and Pop2 target the degradation of the substrates Cdc18p and Rum1p as a heterodimeric Pop1/Pop2 complex even though both Pop1 and Pop2 can also form homodimers [41,42]. Thus both homodimers and heterodimers can form active SCF complexes, thereby providing the possibility for combinatorial regulation of SCF activity.

Many CUL3 complex SRSs form homodimers, including Keap1, MEL-26, RhoBTB2, and SPOP [27,46-48]. Nevertheless, the dimerization mechanism that has been reported for CUL3 complexes does not require SRS dimerization, but rather involves physical interaction between an unneddylated CUL3 and a Nedd8 that is covalently bound to another CUL3 [37] (Fig. 2B). The winged-helix B (WH-B) domain in the C-terminus of the unneddylated CUL3 binds to Nedd8. As Nedd8 is conjugated to a lysine residue within the WH-B domain, the same region of both CUL3 proteins is involved in the interaction.

There is, however, conflicting data on the prevalence of Nedd8-cullin-based dimerization. While Wimuttisuk and Singer found that CUL3 with a mutated SRS-binding site still forms dimers *in vivo *(thereby providing evidence for Nedd8-cullin-based interaction) [37], Chew et al. found that CUL3 with a mutated SRS-binding site does not form dimers *in vivo *[36]. Both groups used the same experimental strategy and cell line. The divergent results imply either that Nedd8-cullin-based interaction is the dominant method of dimerization, or that it has at most a minor role in CUL3 dimerization (and that SRS-based dimerization is predominant). Thus the importance of the Nedd8-cullin binding mechanism is currently unresolved.

Do other cullins besides CUL3 form Nedd8-cullin dimers? It has been observed that human CUL1 in which the adaptor-binding region has been mutated can still form dimers or multimers *in vivo*, suggesting an SRS-independent interaction mechanism [36]. In contrast, the dimerization of budding yeast SCF^Cdc4 ^occurs exclusively through an SRS-mediated mechanism [38]. Moreover, in budding yeast, Nedd8-cullin interaction is unlikely to be an important dimerization pathway, as the Nedd8 ortholog Rub1 is not required for viability, and so cannot be essential for cullin functions [49,50]. It should be noted that budding yeast do not have a clear CUL3 ortholog [9], and it is possible that Nedd8-cullin dimerization is specific for CUL3.

One of the characteristics of the Nedd8-cullin dimerization mechanism is that the dimeric CRL complex must have equal levels of neddylated and unneddylated cullins. Immunoprecipitation of the CUL3 substrate cyclin E pulls down roughly equivalent levels of neddylated and unneddylated CUL3 [37]. In contrast, immunoprecipitation of the substrates of CRL2^VHL ^or SCF^βTrCP ^pulls down predominantly neddylated cullins, implying that SCF^βTrCP ^and CRL2^VHL ^do not function as Nedd8-cullin dimers [13,51,52]. These results suggest that Nedd8-cullin dimerization is not widespread among other (non-CRL3) classes of CRL complexes.

It has been reported that the CRL2 SRS VHL (von Hippel-Lindau tumor suppressor protein) is a dimer *in vivo *and that the dimerization is required for CRL2^VHL ^activity *in vivo *[53]. However, it has also been reported that CUL2 is not present as a dimer or multimer in cells [36]. A model that incorporates both of these results is that a monomeric CRL2 complex binds to dimeric VHL (Fig. 2C). There are currently no published experiments that directly test this model.

### The turnover of substrate-recognition subunits

SRSs recognize and recruit substrates to the CRL complex. Genetic evidence from yeast suggests that F-box proteins compete with each other for binding to the core CRL complex [54,55]. Therefore the regulation of SRS levels (through synthesis or turnover) can directly influence the relative proportion of different CRL complexes.

In both yeast and mammals, F-box proteins are often unstable and undergo proteasome-mediated degradation as a result of autoubiquitination when linked to the SCF complex [55-60]. The overexpression of substrates can stabilize F-box proteins because the bound substrate protects the F-box protein from autoubiquitination [56,60]. Autoubiquitination of SRSs is potentially a broadly based mechanism among CRLs, as it is also observed for the CUL3 complex SRSs RhoBTB2 and Keap1 in mammals, and Btb3 in fission yeast [26,48,61].

In contrast to SCF SRSs, which are often destabilized after binding the SCF complex, the CUL2 complex SRS VHL is stabilized by its association with the CRL2 complex [62,63]. In the absence of binding the CRL2 complex, VHL is degraded through a proteasome-dependent mechanism, presumably via the activity of another E3 [63]. Many other SRS proteins are also degraded through the activity of other E3s. For example, the APC/C (anaphase promoting complex/cyclosome) ubiquitin ligase targets the degradation of the SCF SRSs Skp2 and Tome1, and SCF^βTrCP ^targets the degradation of the SCF SRS Emi1 [64-68].

As we shall see in the following sections, a central role of two major CRL regulators (CSN and CAND1) is to regulate the autoubiquitination of SRSs. Uncontrolled autoubiquitination leads to the inactivation of CRLs due to a loss of SRSs. On the other hand, SRS turnover is essential to allow the switching of SRSs among core CRL complexes so that the relative proportions of different CRLs reflect changes in SRS levels.

### Regulation of CRLs by Nedd8 conjugation

Cullins are post-translationally modified by the covalent attachment of the ubiquitin-like protein Nedd8 to a conserved lysine residue in a process termed neddylation [69]. Nedd8 conjugation increases CRL ubiquitin ligase activity *in vitro *[52,70-72] by promoting the recruitment of the E2 through direct interaction between Nedd8 and the E2 [13,73]. Based on the interaction of E2s with RING finger domains (such as is found in Rbx1) [74], it has been proposed that both Nedd8 and Rbx1 form a common interface for loading the E2 [73]. Nedd8 conjugation is required for the *in vivo *function of CUL1, CUL2, and CUL3 in a number of metazoan species and fission yeast [75-78]. However, in budding yeast, Nedd8 is not essential for SCF-mediated processes, although it does enhance SCF activity [49,50].

The neddylation reaction is similar to the ubiquitination reaction, and involves a heterodimeric E1 (APP-BP-1/Uba3) that activates Nedd8, the E2 UBC12 that conjugates Nedd8 to the cullin, and DCN1 (defective in cullin neddylation) and Rbx1 as E3s [50,51,79-84]. DCN1 was identified as a protein that promotes the neddylation of CUL-3 in *C. elegans *and Cdc53 in budding yeast [80]. DCN1 binds to the cullin and the neddylation E2 UBC12 to facilitate UBC12 loading onto the cullin [81]. While DCN1 promotes neddylation, it is not essential for the neddylation reaction *in vivo *[80]. The CRL component Rbx1 also plays a central role in neddylation. *In vivo*, only cullins that are complexed with Rbx1 undergo neddylation [51,82-84], and mutation of the RING finger motif of Rbx1 abolishes neddylation *in vitro *[82]. Rbx1 can promote neddylation *in vitro *in the absence of DCN1 if there are sufficiently high levels of E2, while the presence of DCN1 allows neddylation at lower E2 levels [81]. Based on the observation that DCN1 can physically bind to Rbx1 [85], it is likely that the two proteins form a multisubunit E3 for the neddylation reaction, although it is possible that Rbx1 is the predominant E3 and DCN1 is a cofactor.

In *C. elegans*, loss of DCN-1 causes embryonic arrest due to loss of CUL-3 activity; while in budding yeast, a *DCN1 *null mutant is viable, consistent with the observation that Rub1 (Nedd8) is not essential in budding yeast [80]. A loss-of-function mutant of an *Arabidopsis Dcn1 *homolog had no effect on SCF^TIR1^-regulated pathways, however, there may be redundancy as there are three *Dcn1*-related genes in *Arabidopsis *[86]. The mammalian DCN1 ortholog (SCCRO, squamous cell carcinoma-related oncogene) is amplified in several human tumors, and functions as an oncogene when overexpressed [87], however there are currently no reports on its role in regulating neddylation.

### Regulation of CRLs by the CSN complex

The COP9 Signalosome (CSN) is a conserved eight-subunit complex that was originally identified in *Arabidopsis *[88,89]. The eight subunits of the CSN complex are homologous to eight subunits of the 19S proteasome lid complex and to three subunits of the eIF3 translation initiation factor complex, suggesting a common origin for these three protein complexes [90]. CSN physically associates with the 26S proteasome, and may function as an alternate lid for the proteasome [91,92]. CSN has been implicated in wide range of biological processes including plant photomorphogenesis, yeast mating pathways, signal transduction, the regulation of DNA repair, and cell cycle regulation [93,94]. Biochemically CSN is associated with three activities, phosphorylation, deneddylation, and deubiquitination, with the latter two activities directly regulating CRLs [93,94].

Nedd8 conjugates are removed from cullins (in a process termed deneddylation) by the isopeptidase activity of the metalloprotease CSN5/Jab1 subunit of CSN [95,96]. Inactivation of CSN increases the levels of neddylated cullins *in vivo *[78,95,97,98]. Counterintuitively, CSN inactivation reduces the activity of CUL1, CUL3, and CUL4-based CRL complexes in cells despite increased neddylation levels [29,78,96,97,99-103]. The loss of CRL activity can be attributed to significantly lower SRS levels due to increased autoubiquitination of SRSs (as shown in yeast, humans, *Drosophila*, and *Neurospora*) [36,103-107]. The deneddylation activity of CSN is primarily responsible for preventing the autoubiquitination of SRSs [107].

The deubiquitinase activity of CSN contributes to the stabilization of CUL1 and CUL3 SRSs in fission yeast, presumably by removing ubiquitin that is conjugated to the SRSs [103,104]. CSN deubiquitinase activity also stabilizes Rbx1 in humans [108,109]. In addition to stabilizing SRSs and Rbx1, CSN is also required for the stability of the cullins CUL1 and CUL3 in *Drosophila*, and CUL1 in *Neurospora *[105,106]. In humans, inactivation of CSN does not affect cullin levels, except for a modest reduction in CUL2 [107].

How the interaction of CSN with CRLs is regulated is unknown. However, the interaction can clearly be subject to active regulation as shown by the rapid release of the CRL4^DDB2 ^complex from CSN upon UV irradiation, and conversely, the rapid binding of the CRL4^CSA ^complex to CSN upon UV irradiation (both CRL4^DDB2 ^and CRL4^CSA ^are involved in aspects of DNA damage repair) [29]. More generally, substrate binding has been implicated in the regulation of neddylation and deneddylation. Substrate binding increases the neddylation levels of human CUL1, CUL2, CUL3, and CUL4 *in vivo *[51,110,111]. *In vitro *experiments indicate that substrate binding increases neddylation levels by preventing the deneddylation of cullins by CSN [110]. Substrate binding presumably blocks deneddylation either by inhibiting the deneddylation of CRLs that are bound to CSN or by preventing the association of CRLs with CSN. In contrast to the *in vitro *results, *in vivo *experiments indicate that substrate binding to CUL1 can increase neddylation levels independently of CSN, suggesting that substrate binding promotes the neddylation reaction in cells [111].

### Regulation of CRLs by the inhibitor CAND1

**Figure 4:**
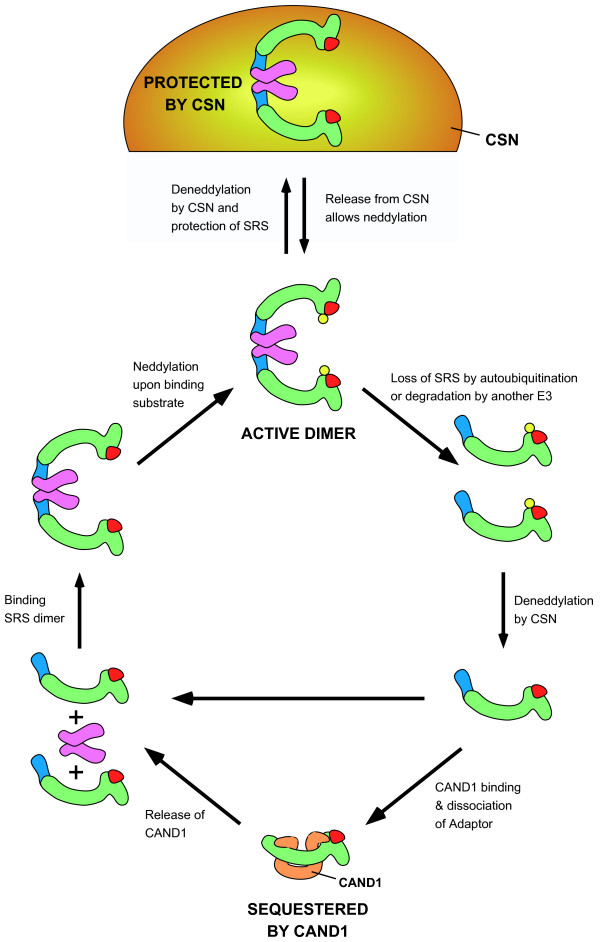
**Proposed activation cycle for an SCF complex**. Diagram of a proposed SCF activation cycle. The SCF complex can shift between an active dimeric complex and a CSN-bound state in which the cullin is deneddylated and the SRS is protected from autoubiquitination (top). The mechanisms that regulate SCF interaction with CSN are not fully understood, but substrate binding may be associated with either releasing SCF from CSN or preventing SCF binding to CSN. When substrate is lacking, SCF complexes can either rebind CSN or lose their SRS due to autodegradation. Loss of the SRS (by autoubiquitination or the activity of other E3 ligases) allows deneddylation by the CSN complex. The deneddylated adaptor-cullin-Rbx1 complex can then either rebind an SRS to reform an SCF complex (horizontal arrow) or undergo sequestration by CAND1 (bottom), in which the adaptor is stripped away from cullin-Rbx1 in the process of CAND1 binding. CAND1 is released via an as yet undefined mechanism that involves cullin-Rbx1 binding either to the adaptor (shown) or an adaptor-SRS complex (not shown). The adaptor-cullin-Rbx1 complex binds an SRS dimer to form a dimeric SCF complex. Substrate binding promotes cullin neddylation to allow full activation of the SCF complex. Proteins are labeled as in Figs 1 and 2.

TIP120A/CAND1 (cullin-associated and neddylation-dissociated) is an inhibitor that binds to cullin-Rbx complexes that lack both neddylation and adaptors [112-115]. CAND1 is a 120 kDa protein composed of multiple HEAT repeats. The crystal structure of human CAND1 bound to a CUL1-Rbx1 complex indicates that CAND1 wraps around the cullin, with the CAND1 N-terminus bound to the cullin C-terminus and the CAND1 C-terminus bound to the cullin N-terminus [116] (Fig. 4). CAND1 binding blocks both the adaptor binding site and the Nedd8 conjugation site.

CAND1 is capable of binding to all cullins in human cells [112,114]. However, in certain cells, CAND1 preferentially associates with a subset of cullins. In human HEK293T cells, CAND1 associates primarily with CUL1 [111,115]. CAND1 can also bind to CUL4A and CUL5 in HEK293T cells, but there is no observed interaction with CUL2 or CUL3 [112]. In contrast, in human HeLa cells, CAND1 interacts with CUL1, CUL2, CUL3, and CUL4A [114]. The reason for these differences (either based on cell lines or experimental conditions) is not understood. In *C. elegans*, CAND1 binds at high level to CUL-2, but does not have detectable binding to CUL-3 [117,118].

CAND1 binding to cullin-Rbx is incompatible with neddylation. The presence of Nedd8 on the cullin blocks CAND1 binding, suggesting that CAND1 binds to cullin-Rbx only after CSN has removed Nedd8 [112,113]. CAND1 can dissociate the adaptor Skp1 from unneddylated CUL1 *in vitro*, suggesting that once Nedd8 has been removed, CAND1 is capable of stripping off the adaptor and binding the cullin [112] (Fig. 4).

Counterintuitively, inactivation of CAND1 leads to the inactivation of SCF complexes in humans and *Arabidopsis*, and CUL3 complexes in humans [36,61,113,119-121]. In the case of human SCF^Skp2^, the inactivation of CAND1 is correlated with reduced levels of the SRS Skp2, which is proposed to result from autoubiquitination [36,113]. In contrast, the activity of the CRL3^Keap1 ^complex is inhibited upon CAND1 inactivation even though increased levels of Keap1 bind to CUL3, and Keap1 interaction with its substrate is increased, suggesting that the presence of CAND1 is required for CRL3^Keap1 ^activity independently of SRS stabilization [61].

It is reasonable to assume that cells do not produce CAND1 in order to permanently sequester cullin-Rbx complexes, as this would be energetically wasteful. It is therefore pertinent to ask how cullin-Rbx is released from CAND1. There are two potential mechanisms that have been tested to address CAND1 dissociation, the first is neddylation, and the second is the binding of additional CRL components. Neddylation was initially shown to dissociate CAND1 based on *in vitro *experiments with endogenous human CUL1 that was bound to antibody after immunoprecipitation [112]. However, studies using soluble, recombinant CUL1 showed that CAND1 is not dissociated by neddylation and instead completely blocks access to the neddylation site [116,122]. It should be noted that these experiments used different sources of CUL1, endogenous and recombinant (see below).

The second mechanism for CAND1 dissociation is the binding of CRL components. Two groups obtained somewhat different results for this mechanism. Zheng et al. reported that CAND1 could be dissociated from endogenous CUL1 by the addition of the adaptor Skp1 and ATP [113]. However, Bornstein et al. indicated that the Skp1-Skp2 complex (but not Skp1 alone) could dissociate CAND1 from endogenous CUL1, and that ATP had no effect on the dissociation [110]. It is currently unclear whether adaptor-SRS or adaptor alone is involved in the release of cullin-Rbx. Nevertheless, it is significant that Bornstein et al. showed that Skp1-Skp2 could dissociate CAND1 from endogenous CUL1 but not from recombinant CUL1 [110]. The finding that endogenous CUL1 is more easily released from CAND1 implies a role for either critical post-translationally modification(s) of CUL1 or a 'dissociation factor'.

It has recently been reported that co-inactivation of murine c-Abl and the related c-Arg tyrosine kinase is associated with increased binding between CUL4A and CAND1 [123]. This suggests that murine c-Abl and c-Arg either promote the dissociation of CAND1 from CUL4A or prevent their association. The mechanistic pathway(s) by which these kinases regulate this interaction has not been resolved.

### Potential crosstalk between CAND1 and CSN

Is there crosstalk between CAND1 and CSN in the regulation of CRLs? *In vitro*, CAND1 increases the CSN-mediated deneddylation of the SCF complex [124]. *In vivo*, inactivation of CAND1 slows the deneddylation rate (when the neddylation system is inactivated), suggesting that CAND1 promotes deneddylation [36]. Because CAND1 has not been observed to physically interact with CSN, it has been proposed that CAND1 indirectly facilitates deneddylation by binding to unneddylated cullins with high affinity, thereby shifting CSN interactions towards neddylated cullins [124]. However, it is still possible that CAND1 plays a more direct role by stripping unneddylated cullins from the CSN complex. It should be noted that siRNA depletion of human CAND1 does not appreciably increase the neddylation levels of CUL1, suggesting that CAND1 is not rate limiting for determining steady state neddylation levels in human cells [36,113].

A newly discovered CRL interactor, SAP130/SF3b-3, has the potential to provide feedback links between CSN and CAND1 [125]. SAP130 is a DDB1-related protein that is a component of the 17S U2 snRNP particle and the STAGA and TFTC transcription complexes [126-128]. SAP130 physically associates with CSN, and also binds to complete SCF, CRL2, and CRL4 complexes via direct interaction with the cullins [125]. SAP130 associates almost exclusively with neddylated cullins. However, inactivation of CAND1 increases the overall level of CUL2 that binds to SAP130 (as well as the proportion of unneddylated CUL2 that is bound) [125]. Therefore, it appears that the sequestration of cullins by CAND1 limits their interaction with SAP130. Currently, the role of SAP130 in CRL regulation is unclear, and ectopic expression or moderate knockdown of SAP130 does not affect cullin neddylation levels [125]. Nevertheless, SAP130's interaction with CSN and the regulation of its cullin binding by CAND1 suggest that SAP130 may provide a functional link between CSN and CAND1.

### CRL activation cycles

CRLs transit through different stages of assembly, sequestration, and neddylation. These changes can be considered an activation cycle, with CRL components switching from an inactive form (lacking Nedd8 and/or adaptor or SRS, and potentially sequestered by CAND1) to an active form (with attached SRS and Nedd8 conjugation). An outline of a proposed CRL activation cycle is presented in Figure 4.

#### CSN-mediated CRL protection

There appears to be two pathways by which CRLs can switch between active and inactive forms. One pathway involves CRL docking with CSN (Fig. 4, top). CSN can bind to completely assembled CUL1 and CUL4 CRL complexes, based on the observation that all CRL components, including SRSs, are found to associate with CSN [29,95,97,99,101,129,130]. The deneddylation and deubiquitination activities of CSN can stabilize SRSs by preventing autoubiquitination [36,103-107]. CSN therefore keeps CRL complexes in a protected, inactive state. What regulates CRL binding to CSN is not fully understood. Substrate binding to SCF complexes is incompatible with CSN-mediated deneddylation [110], and it is possible that substrate binding leads to the dissociation of CRL complexes from CSN or inhibits the association of CRLs with CSN. Once CRL complexes are released from CSN, they can become neddylated and fully active. The depletion of substrates may lead to the re-association of CRLs with CSN, although this has not yet been experimentally demonstrated.

#### CAND1-mediated CRL sequestration

The second pathway to modulate CRL activity is initiated by the degradation of the SRS (Fig. 4). In the absence of substrate, SRSs can undergo autoubiquitination [56,60]. Additionally, other E3 ligases can induce SRS degradation. Once the SRS is degraded, the core CRL components can associate with CSN and undergo deneddylation. CAND1 can presumably dissociate adaptors from the unneddylated cullin-Rbx complex *in vivo*, as CAND1 has been shown capable of doing so *in vitro *[112]. The mechanism by which cullin-Rbx complexes are released from CAND1 sequestration has not yet been resolved. However, once released, the binding of cullin-Rbx to adaptor and SRS will reconstitute the CRL complex. The binding of substrate then induces neddylation and full activity [111].

### The purposes of the activation cycle

What is the purpose of the activation cycle for CRLs? There are three major possibilities. The first purpose appears to be to allow CRLs to efficiently switch between different SRSs. SRS degradation frees the core CRL components to reassemble with new SRSs. A dynamic CRL activation cycle allows adjustments in the proportions of specific CRL complexes in order to reflect changes in the cellular levels of SRSs. It is currently unclear whether CAND1 sequestration is a common aspect of SRS switching or if CRL components sans-SRS generally bypass this step (Fig. 4). The observation that only certain cullins interact appreciably with CAND1 in certain mammalian cell lines suggests that CAND1 sequestration is not a requirement for SRS switching.

The second purpose of the activation cycle is to stabilize CRL complexes. Loss of either CSN or CAND1 produces a loss of CRL activity that is attributable, in large part, to the autodegradation of SRSs [36,103-107,113]. This suggests that both CSN and CAND1 are essential to dampen uncontrolled CRL ubiquitin ligase activity in order to prevent CRLs from "burning out" by autoubiquitination of the available pool of SRSs.

The third potential purpose is that cycles of neddylation and deneddylation are directly required for CRL ubiquitin ligase activity. This model is based largely on studies of CUL-3 in *C. elegans *[78]. *C. elegans *CUL-3 is inactive when either neddylation or deneddylation pathways are compromised, yet combining compromised neddylation and deneddylation pathways restores CUL-3 function [78]. This suggested that balanced (but slower) cycling between neddylated and unneddylated states allows CUL-3 activity, while unchecked neddylation or deneddylation (which eliminates cycling) is incompatible with CUL-3 activity. However, an alternative interpretation of the results has been proposed that casts doubt on this model [37]. CUL-3 dimers created by Nedd8-cullin interaction require both neddylated and unneddylated CUL3 in equal proportion [37] (Fig. 2B). Inactivation of either the neddylation or deneddylation pathways by themselves would produce predominantly unneddylated or neddylated CUL-3, respectively. In such a situation, the absence of sufficient levels of both neddylated and unneddylated CUL-3 would reduce the formation of active Nedd8-cullin dimers. Therefore, until there is additional evidence, it is not possible to conclude that neddylation/deneddylation cycles are inherently required for CRL activity.

### Unresolved Questions

There are unresolved questions about multiple aspects of the global regulation of CRLs. Dimerization has only recently been recognized as an essential characteristic of many CRLs. It is not yet known to what extent the different dimerization mechanisms are utilized. The SRS-based dimerization mechanism is well established for SCF complexes but has not been rigorously tested for other cullin-based CRLs. Conversely, the Nedd8-cullin dimerization mechanism has so far only been reported for CUL3 CRL complexes, and the structure has not been fully determined. Finally, the possibility of monomeric CRL cores binding to dimeric SRSs has not yet been rigorously tested.

While the biochemistry of cullin neddylation has been determined, it is not yet clear how neddylation is regulated *in vivo*. There is evidence that substrate binding promotes neddylation, yet how substrate binding mechanistically induces neddylation is not apparent. There is also evidence that substrate binding inhibits deneddylation by CSN, but it is unclear if this works by directly blocking deneddylation or by blocking association with CSN. Overall, what regulates the association of CRLs with CSN and the dissociation of CRLs from CSN is not well understood.

The functional role of CAND1 in sequestering cullins is still mysterious. CAND1 is important to prevent SRS autoubiquitination, but so is CSN, and it is unclear why CAND1 is required in addition to CSN to prevent autoubiquitination. Additionally, multiple aspects of CAND1 activity are unclear. CAND1 only binds to unneddylated cullins, but it is not known whether CAND1 binding is actively coupled to CSN deneddylation, as is suggested by *in vitro *experiments. It is also not known how CAND1 is released from cullin-Rbx in cells. The observation that endogenous cullins can be released from CAND1 while recombinant cullins cannot, suggests either that the cullin must be post-translationally modified or that a 'dissociation factor' is required to release CAND1. Finally, it is unclear why CAND1 exhibits preferential binding to particular classes of cullins in different cell lines and organisms.

The activation cycle is not fully understood. It would be helpful to know which stages of the cycle are rate limiting and accumulate CRL components during steady-state conditions. It also remains to be determined whether different classes of CRLs employ inherently different activation cycles. With so many fundamental questions still remaining, it is likely that the study of the regulation of CRL complexes will continue to be an interesting and productive area of research.

## References

[B1] Ciechanover A, Finley D, Varshavsky A (1984). Ubiquitin dependence of selective protein degradation demonstrated in the mammalian cell cycle mutant ts85. Cell.

[B2] Rock KL, Gramm C, Rothstein L, Clark K, Stein R, Dick L, Hwang D, Goldberg AL (1994). Inhibitors of the proteasome block the degradation of most cell proteins and the generation of peptides presented on MHC class I molecules. Cell.

[B3] Glickman MH, Ciechanover A (2002). The ubiquitin-proteasome proteolytic pathway: destruction for the sake of construction. Physiol Rev.

[B4] Pickart CM (2001). Mechanisms underlying ubiquitination. Annu Rev Biochem.

[B5] Hicke L (2001). Protein regulation by monoubiquitin. Nat Rev Mol Cell Biol.

[B6] Pickart CM, Fushman D (2004). Polyubiquitin chains: polymeric protein signals. Curr Opin Chem Biol.

[B7] Petroski MD, Deshaies RJ (2005). Function and regulation of cullin-RING ubiquitin ligases. Nat Rev Mol Cell Biol.

[B8] Kipreos ET, Lander LE, Wing JP, He WW, Hedgecock EM (1996). cul-1 is required for cell cycle exit in C. elegans and identifies a novel gene family. Cell.

[B9] Nayak S, Santiago FE, Jin H, Lin D, Schedl T, Kipreos ET (2002). The Caenorhabditis elegans Skp1-related gene family: diverse functions in cell proliferation, morphogenesis, and meiosis. Curr Biol.

[B10] Skaar JR, Florens L, Tsutsumi T, Arai T, Tron A, Swanson SK, Washburn MP, DeCaprio JA (2007). PARC and CUL7 form atypical cullin RING ligase complexes. Cancer Res.

[B11] Zheng N, Schulman BA, Song L, Miller JJ, Jeffrey PD, Wang P, Chu C, Koepp DM, Elledge SJ, Pagano M, Conaway RC, Conaway JW, Harper JW, Pavletich NP (2002). Structure of the Cul1-Rbx1-Skp1-F boxSkp2 SCF ubiquitin ligase complex. Nature.

[B12] Wu G, Xu G, Schulman BA, Jeffrey PD, Harper JW, Pavletich NP (2003). Structure of a beta-TrCP1-Skp1-beta-catenin complex: destruction motif binding and lysine specificity of the SCF(beta-TrCP1) ubiquitin ligase. Mol Cell.

[B13] Kawakami T, Chiba T, Suzuki T, Iwai K, Yamanaka K, Minato N, Suzuki H, Shimbara N, Hidaka Y, Osaka F, Omata M, Tanaka K (2001). NEDD8 recruits E2-ubiquitin to SCF E3 ligase. EMBO J.

[B14] Kipreos ET, Pagano M (2000). The F-box protein family. Genome Biol.

[B15] Jin J, Cardozo T, Lovering RC, Elledge SJ, Pagano M, Harper JW (2004). Systematic analysis and nomenclature of mammalian F-box proteins. Genes Dev.

[B16] Kus BM, Caldon CE, Andorn-Broza R, Edwards AM (2004). Functional interaction of 13 yeast SCF complexes with a set of yeast E2 enzymes in vitro. Proteins.

[B17] Cenciarelli C, Chiaur DS, Guardavaccaro D, Parks W, Vidal M, Pagano M (1999). Identification of a family of human F-box proteins. Curr Biol.

[B18] Kamura T, Koepp DM, Conrad MN, Skowyra D, Moreland RJ, Iliopoulos O, Lane WS, Kaelin WG, Elledge SJ, Conaway RC, Harper JW, Conaway JW (1999). Rbx1, a component of the VHL tumor suppressor complex and SCF ubiquitin ligase. Science.

[B19] Pause A, Peterson B, Schaffar G, Stearman R, Klausner RD (1999). Studying interactions of four proteins in the yeast two-hybrid system: structural resemblance of the pVHL/elongin BC/hCUL-2 complex with the ubiquitin ligase complex SKP1/cullin/F-box protein. Proc Natl Acad Sci USA.

[B20] Conaway RC, Conaway JW (2002). The von Hippel-Lindau tumor suppressor complex and regulation of hypoxia-inducible transcription. Adv Cancer Res.

[B21] Kamura T, Maenaka K, Kotoshiba S, Matsumoto M, Kohda D, Conaway RC, Conaway JW, Nakayama KI (2004). VHL-box and SOCS-box domains determine binding specificity for Cul2-Rbx1 and Cul5-Rbx2 modules of ubiquitin ligases. Genes Dev.

[B22] Yu Y, Xiao Z, Ehrlich ES, Yu X, Yu XF (2004). Selective assembly of HIV-1 Vif-Cul5-ElonginB-ElonginC E3 ubiquitin ligase complex through a novel SOCS box and upstream cysteines. Genes Dev.

[B23] Mahrour N, Redwine WB, Florens L, Swanson SK, Martin-Brown S, Bradford WD, Staehling-Hampton K, Washburn MP, Conaway RC, Conaway JW (2008). Characterization of cullin-box sequences that direct recruitment of Cul2-Rbx1 and Cul5-Rbx2 modules to elongin BC-based ubiquitin ligases. J Biol Chem.

[B24] Kohroki J, Nishiyama T, Nakamura T, Masuho Y (2005). ASB proteins interact with Cullin5 and Rbx2 to form E3 ubiquitin ligase complexes. FEBS Lett.

[B25] Furukawa M, He YJ, Borchers C, Xiong Y (2003). Targeting of protein ubiquitination by BTB-Cullin 3-Roc1 ubiquitin ligases. Nat Cell Biol.

[B26] Geyer R, Wee S, Anderson S, Yates J, Wolf DA (2003). BTB/POZ domain proteins are putative substrate adaptors for cullin 3 ubiquitin ligases. Mol Cell.

[B27] Pintard L, Willis JH, Willems A, Johnson JL, Srayko M, Kurz T, Glaser S, Mains PE, Tyers M, Bowerman B, Peter M (2003). The BTB protein MEL-26 is a substrate-specific adaptor of the CUL-3 ubiquitin-ligase. Nature.

[B28] Xu L, Wei Y, Reboul J, Vaglio P, Shin TH, Vidal M, Elledge SJ, Harper JW (2003). BTB proteins are substrate-specific adaptors in an SCF-like modular ubiquitin ligase containing CUL-3. Nature.

[B29] Groisman R, Polanowska J, Kuraoka I, Sawada J, Saijo M, Drapkin R, Kisselev AF, Tanaka K, Nakatani Y (2003). The ubiquitin ligase activity in the DDB2 and CSA complexes is differentially regulated by the COP9 signalosome in response to DNA damage. Cell.

[B30] Wertz IE, O'Rourke KM, Zhang Z, Dornan D, Arnott D, Deshaies RJ, Dixit VM (2004). Human De-etiolated-1 regulates c-Jun by assembling a CUL4A ubiquitin ligase. Science.

[B31] Angers S, Li T, Yi X, MacCoss MJ, Moon RT, Zheng N (2006). Molecular architecture and assembly of the DDB1-CUL4A ubiquitin ligase machinery. Nature.

[B32] Higa LA, Wu M, Ye T, Kobayashi R, Sun H, Zhang H (2006). CUL4-DDB1 ubiquitin ligase interacts with multiple WD40-repeat proteins and regulates histone methylation. Nat Cell Biol.

[B33] He YJ, McCall CM, Hu J, Zeng Y, Xiong Y (2006). DDB1 functions as a linker to recruit receptor WD40 proteins to CUL4-ROC1 ubiquitin ligases. Genes Dev.

[B34] Jin J, Arias EE, Chen J, Harper JW, Walter JC (2006). A family of diverse Cul4-Ddb1-interacting proteins includes Cdt2, which is required for S phase destruction of the replication factor Cdt1. Mol Cell.

[B35] Hu J, McCall CM, Ohta T, Xiong Y (2004). Targeted ubiquitination of CDT1 by the DDB1-CUL4A-ROC1 ligase in response to DNA damage. Nat Cell Biol.

[B36] Chew EH, Poobalasingam T, Hawkey CJ, Hagen T (2007). Characterization of cullin-based E3 ubiquitin ligases in intact mammalian cells – evidence for cullin dimerization. Cell Signal.

[B37] Wimuttisuk W, Singer JD (2006). The Cullin3 Ubiquitin Ligase Functions as a Nedd8-bound Heterodimer. Mol Biol Cell.

[B38] Tang X, Orlicky S, Lin Z, Willems A, Neculai D, Ceccarelli D, Mercurio F, Shilton BH, Sicheri F, Tyers M (2007). Suprafacial orientation of the SCFCdc4 dimer accommodates multiple geometries for substrate ubiquitination. Cell.

[B39] Suzuki H, Chiba T, Suzuki T, Fujita T, Ikenoue T, Omata M, Furuichi K, Shikama H, Tanaka K (2000). Homodimer of two F-box proteins betaTrCP1 or betaTrCP2 binds to IkappaBalpha for signal-dependent ubiquitination. J Biol Chem.

[B40] Dixon C, Brunson LE, Roy MM, Smothers D, Sehorn MG, Mathias N (2003). Overproduction of polypeptides corresponding to the amino terminus of the F-box proteins Cdc4p and Met30p inhibits ubiquitin ligase activities of their SCF complexes. Eukaryot Cell.

[B41] Kominami K, Ochotorena I, Toda T (1998). Two F-box/WD-repeat proteins Pop1 and Pop2 form hetero- and homo-complexes together with cullin-1 in the fission yeast SCF (Skp1-Cullin-1-F-box) ubiquitin ligase. Genes Cells.

[B42] Wolf DA, McKeon F, Jackson PK (1999). F-box/WD-repeat proteins pop1p and Sud1p/Pop2p form complexes that bind and direct the proteolysis of cdc18p. Curr Biol.

[B43] Welcker M, Clurman BE (2007). Fbw7/hCDC4 dimerization regulates its substrate interactions. Cell division.

[B44] Hao B, Oehlmann S, Sowa ME, Harper JW, Pavletich NP (2007). Structure of a Fbw7-Skp1-cyclin E complex: multisite-phosphorylated substrate recognition by SCF ubiquitin ligases. Mol Cell.

[B45] Zhang W, Koepp DM (2006). Fbw7 isoform interaction contributes to cyclin E proteolysis. Mol Cancer Res.

[B46] McMahon M, Thomas N, Itoh K, Yamamoto M, Hayes JD (2006). Dimerization of substrate adaptors can facilitate cullin-mediated ubiquitylation of proteins by a "tethering" mechanism: a two-site interaction model for the Nrf2-Keap1 complex. J Biol Chem.

[B47] Hernandez-Munoz I, Lund AH, van der Stoop P, Boutsma E, Muijrers I, Verhoeven E, Nusinow DA, Panning B, Marahrens Y, van Lohuizen M (2005). Stable X chromosome inactivation involves the PRC1 Polycomb complex and requires histone MACROH2A1 and the CULLIN3/SPOP ubiquitin E3 ligase. Proc Natl Acad Sci USA.

[B48] Wilkins A, Ping Q, Carpenter CL (2004). RhoBTB2 is a substrate of the mammalian Cul3 ubiquitin ligase complex. Genes Dev.

[B49] Lammer D, Mathias N, Laplaza JM, Jiang W, Liu Y, Callis J, Goebl M, Estelle M (1998). Modification of yeast Cdc53p by the ubiquitin-related protein rub1p affects function of the SCFCdc4 complex. Genes Dev.

[B50] Liakopoulos D, Doenges G, Matuschewski K, Jentsch S (1998). A novel protein modification pathway related to the ubiquitin system. Embo J.

[B51] Sufan RI, Ohh M (2006). Role of the NEDD8 modification of Cul2 in the sequential activation of ECV complex. Neoplasia.

[B52] Read MA, Brownell JE, Gladysheva TB, Hottelet M, Parent LA, Coggins MB, Pierce JW, Podust VN, Luo RS, Chau V, Palombella VJ (2000). Nedd8 modification of cul-1 activates SCF(beta(TrCP))-dependent ubiquitination of IkappaBalpha. Mol Cell Biol.

[B53] Chung J, Roberts AM, Chow J, Coady-Osberg N, Ohh M (2006). Homotypic association between tumour-associated VHL proteins leads to the restoration of HIF pathway. Oncogene.

[B54] Patton EE, Willems AR, Sa D, Kuras L, Thomas D, Craig KL, Tyers M (1998). Cdc53 is a scaffold protein for multiple Cdc34/Skp1/F-box protein complexes that regulate cell division and methionine biosynthesis in yeast. Genes & Dev.

[B55] Zhou P, Howley PM (1998). Ubiquitination and degradation of the substrate recognition subunits of SCF ubiquitin-protein ligases. Mol Cell.

[B56] Galan JM, Peter M (1999). Ubiquitin-dependent degradation of multiple F-box proteins by an autocatalytic mechanism. Proc Natl Acad Sci USA.

[B57] Mathias N, Johnson S, Byers B, Goebl M (1999). The abundance of cell cycle regulatory protein Cdc4p is controlled by interactions between its F box and Skp1p. Mol Cell Biol.

[B58] Smothers DB, Kozubowski L, Dixon C, Goebl MG, Mathias N (2000). The abundance of Met30p limits SCF(Met30p) complex activity and is regulated by methionine availability. Mol Cell Biol.

[B59] Wirbelauer C, Sutterluty H, Blondel M, Gstaiger M, Peter M, Reymond F, Krek W (2000). The F-box protein Skp2 is a ubiquitylation target of a Cul1-based core ubiquitin ligase complex: evidence for a role of Cul1 in the suppression of Skp2 expression in quiescent fibroblasts. EMBO J.

[B60] Li Y, Gazdoiu S, Pan ZQ, Fuchs SY (2004). Stability of homologue of Slimb F-box protein is regulated by availability of its substrate. J Biol Chem.

[B61] Lo SC, Hannink M (2006). CAND1-mediated substrate adaptor recycling is required for efficient repression of Nrf2 by Keap1. Mol Cell Biol.

[B62] Kamura T, Brower CS, Conaway RC, Conaway JW (2002). A molecular basis for stabilization of the von Hippel-Lindau (VHL) tumor suppressor protein by components of the VHL ubiquitin ligase. J Biol Chem.

[B63] Schoenfeld AR, Davidowitz EJ, Burk RD (2000). Elongin BC complex prevents degradation of von Hippel-Lindau tumor suppressor gene products. Proc Natl Acad Sci USA.

[B64] Ayad NG, Rankin S, Murakami M, Jebanathirajah J, Gygi S, Kirschner MW (2003). Tome-1, a trigger of mitotic entry, is degraded during G1 via the APC. Cell.

[B65] Margottin-Goguet F, Hsu JY, Loktev A, Hsieh HM, Reimann JD, Jackson PK (2003). Prophase destruction of Emi1 by the SCF(betaTrCP/Slimb) ubiquitin ligase activates the anaphase promoting complex to allow progression beyond prometaphase. Dev Cell.

[B66] Wei W, Ayad NG, Wan Y, Zhang GJ, Kirschner MW, Kaelin WG (2004). Degradation of the SCF component Skp2 in cell-cycle phase G1 by the anaphase-promoting complex. Nature.

[B67] Bashir T, Dorrello NV, Amador V, Guardavaccaro D, Pagano M (2004). Control of the SCF(Skp2-Cks1) ubiquitin ligase by the APC/C(Cdh1) ubiquitin ligase. Nature.

[B68] Guardavaccaro D, Kudo Y, Boulaire J, Barchi M, Busino L, Donzelli M, Margottin-Goguet F, Jackson PK, Yamasaki L, Pagano M (2003). Control of meiotic and mitotic progression by the F box protein beta-Trcp1 in vivo. Dev Cell.

[B69] Pan ZQ, Kentsis A, Dias DC, Yamoah K, Wu K (2004). Nedd8 on cullin: building an expressway to protein destruction. Oncogene.

[B70] Morimoto M, Nishida T, Honda R, Yasuda H (2000). Modification of cullin-1 by ubiquitin-like protein Nedd8 enhances the activity of SCF(skp2) toward p27(kip1). Biochem Biophys Res Commun.

[B71] Podust VN, Brownell JE, Gladysheva TB, Luo RS, Wang C, Coggins MB, Pierce JW, Lightcap ES, Chau V (2000). A Nedd8 conjugation pathway is essential for proteolytic targeting of p27Kip1 by ubiquitination. Proc Natl Acad Sci USA.

[B72] Wu K, Chen A, Pan ZQ (2000). Conjugation of Nedd8 to CUL1 enhances the ability of the ROC1-CUL1 complex to promote ubiquitin polymerization. J Biol Chem.

[B73] Sakata E, Yamaguchi Y, Miyauchi Y, Iwai K, Chiba T, Saeki Y, Matsuda N, Tanaka K, Kato K (2007). Direct interactions between NEDD8 and ubiquitin E2 conjugating enzymes upregulate cullin-based E3 ligase activity. Nat Struct Mol Biol.

[B74] Zheng N, Wang P, Jeffrey PD, Pavletich NP (2000). Structure of a c-Cbl-UbcH7 complex: RING domain function in ubiquitin-protein ligases. Cell.

[B75] Osaka F, Saeki M, Katayama S, Aida N, Toh EA, Kominami K, Toda T, Suzuki T, Chiba T, Tanaka K, Kato S (2000). Covalent modifier NEDD8 is essential for SCF ubiquitin-ligase in fission yeast. Embo J.

[B76] Ohh M, Kim WY, Moslehi JJ, Chen Y, Chau V, Read MA, Kaelin WG (2002). An intact NEDD8 pathway is required for Cullin-dependent ubiquitylation in mammalian cells. EMBO Rep.

[B77] Ou CY, Lin YF, Chen YJ, Chien CT (2002). Distinct protein degradation mechanisms mediated by Cul1 and Cul3 controlling Ci stability in Drosophila eye development. Genes Dev.

[B78] Pintard L, Kurz T, Glaser S, Willis JH, Peter M, Bowerman B (2003). Neddylation and deneddylation of CUL-3 is required to target MEI-1/Katanin for degradation at the meiosis-to-mitosis transition in C. elegans. Curr Biol.

[B79] Gong L, Yeh ET (1999). Identification of the activating and conjugating enzymes of the NEDD8 conjugation pathway. J Biol Chem.

[B80] Kurz T, Ozlu N, Rudolf F, O'Rourke SM, Luke B, Hofmann K, Hyman AA, Bowerman B, Peter M (2005). The conserved protein DCN-1/Dcn1p is required for cullin neddylation in C. elegans and S. cerevisiae. Nature.

[B81] Kurz T, Chou Y-C, Willems A, Meyer-Schaller N, Hecht M-L, Tyers M, Peter M, Sicheri F (2008). Dcn1 functions as a scaffold-type E3 ligase for cullin neddylation. Mol Cell.

[B82] Kamura T, Conrad MN, Yan Q, Conaway RC, Conaway JW (1999). The Rbx1 subunit of SCF and VHL E3 ubiquitin ligase activates Rub1 modification of cullins Cdc53 and Cul2. Genes Dev.

[B83] Furukawa M, Zhang Y, McCarville J, Ohta T, Xiong Y (2000). The CUL1 C-terminal sequence and ROC1 are required for efficient nuclear accumulation, NEDD8 modification, and ubiquitin ligase activity of CUL1. Mol Cell Biol.

[B84] Megumi Y, Miyauchi Y, Sakurai H, Nobeyama H, Lorick K, Nakamura E, Chiba T, Tanaka K, Weissman AM, Kirisako T, Ogawa O, Iwai K (2005). Multiple roles of Rbx1 in the VBC-Cul2 ubiquitin ligase complex. Genes Cells.

[B85] Yang X, Zhou J, Sun L, Wei Z, Gao J, Gong W, Xu RM, Rao Z, Liu Y (2007). Structural basis for the function of DCN-1 in protein Neddylation. J Biol Chem.

[B86] Biswas KK, Ooura C, Higuchi K, Miyazaki Y, Van Nguyen V, Rahman A, Uchimiya H, Kiyosue T, Koshiba T, Tanaka A, Narumi I, Oono Y (2007). Genetic Characterization of Mutants Resistant to the Antiauxin p-Chlorophenoxyisobutyric Acid Reveals That AAR3, a Gene Encoding a DCN1-Like Protein, Regulates Responses to the Synthetic Auxin 2,4-Dichlorophenoxyacetic Acid in Arabidopsis Roots. Plant Physiol.

[B87] Sarkaria I, P Oc, Talbot SG, Reddy PG, Ngai I, Maghami E, Patel KN, Lee B, Yonekawa Y, Dudas M, Kaufman A, Ryan R, Ghossein R, Rao PH, Stoffel A, Ramanathan Y, Singh B (2006). Squamous cell carcinoma related oncogene/DCUN1D1 is highly conserved and activated by amplification in squamous cell carcinomas. Cancer Res.

[B88] Wei N, Chamovitz DA, Deng XW (1994). Arabidopsis COP9 is a component of a novel signaling complex mediating light control of development. Cell.

[B89] Wei N, Deng XW (1992). COP9: a new genetic locus involved in light-regulated development and gene expression in arabidopsis. Plant Cell.

[B90] Schwechheimer C (2004). The COP9 signalosome (CSN): an evolutionary conserved proteolysis regulator in eukaryotic development. Biochim Biophys Acta.

[B91] Peng Z, Shen Y, Feng S, Wang X, Chitteti BN, Vierstra RD, Deng XW (2003). Evidence for a physical association of the COP9 signalosome, the proteasome, and specific SCF E3 ligases in vivo. Curr Biol.

[B92] Huang X, Hetfeld BK, Seifert U, Kahne T, Kloetzel PM, Naumann M, Bech-Otschir D, Dubiel W (2005). Consequences of COP9 signalosome and 26S proteasome interaction. Febs J.

[B93] Wolf DA, Zhou C, Wee S (2003). The COP9 signalosome: an assembly and maintenance platform for cullin ubiquitin ligases?. Nat Cell Biol.

[B94] Cope GA, Deshaies RJ (2003). COP9 signalosome: a multifunctional regulator of SCF and other cullin-based ubiquitin ligases. Cell.

[B95] Lyapina S, Cope G, Shevchenko A, Serino G, Tsuge T, Zhou C, Wolf DA, Wei N, Deshaies RJ (2001). Promotion of NEDD-CUL1 conjugate cleavage by COP9 signalosome. Science.

[B96] Cope GA, Suh GS, Aravind L, Schwarz SE, Zipursky SL, Koonin EV, Deshaies RJ (2002). Role of predicted metalloprotease motif of Jab1/Csn5 in cleavage of Nedd8 from Cul1. Science.

[B97] Schwechheimer C, Serino G, Callis J, Crosby WL, Lyapina S, Deshaies RJ, Gray WM, Estelle M, Deng XW (2001). Interactions of the COP9 signalosome with the E3 ubiquitin ligase SCFTIRI in mediating auxin response. Science.

[B98] Menon S, Chi H, Zhang H, Deng XW, Flavell RA, Wei N (2007). COP9 signalosome subunit 8 is essential for peripheral T cell homeostasis and antigen receptor-induced entry into the cell cycle from quiescence. Nat Immunol.

[B99] Feng S, Ma L, Wang X, Xie D, Dinesh-Kumar SP, Wei N, Deng XW (2003). The COP9 signalosome interacts physically with SCF COI1 and modulates jasmonate responses. Plant Cell.

[B100] Liu C, Powell KA, Mundt K, Wu L, Carr AM, Caspari T (2003). Cop9/signalosome subunits and Pcu4 regulate ribonucleotide reductase by both checkpoint-dependent and -independent mechanisms. Genes Dev.

[B101] Wang X, Feng S, Nakayama N, Crosby WL, Irish V, Deng XW, Wei N (2003). The COP9 signalosome interacts with SCF UFO and participates in Arabidopsis flower development. Plant Cell.

[B102] Doronkin S, Djagaeva I, Beckendorf SK (2003). The COP9 signalosome promotes degradation of Cyclin E during early Drosophila oogenesis. Dev Cell.

[B103] Zhou C, Wee S, Rhee E, Naumann M, Dubiel W, Wolf DA (2003). Fission yeast COP9/signalosome suppresses cullin activity through recruitment of the deubiquitylating enzyme Ubp12p. Mol Cell.

[B104] Wee S, Geyer RK, Toda T, Wolf DA (2005). CSN facilitates Cullin-RING ubiquitin ligase function by counteracting autocatalytic adapter instability. Nat Cell Biol.

[B105] He Q, Cheng P, He Q, Liu Y (2005). The COP9 signalosome regulates the Neurospora circadian clock by controlling the stability of the SCFFWD-1 complex. Genes Dev.

[B106] Wu JT, Lin HC, Hu YC, Chien CT (2005). Neddylation and deneddylation regulate Cul1 and Cul3 protein accumulation. Nat Cell Biol.

[B107] Cope GA, Deshaies RJ (2006). Targeted silencing of Jab1/Csn5 in human cells downregulates SCF activity through reduction of F-box protein levels. BMC Biochem.

[B108] Hetfeld BK, Helfrich A, Kapelari B, Scheel H, Hofmann K, Guterman A, Glickman M, Schade R, Kloetzel PM, Dubiel W (2005). The zinc finger of the CSN-associated deubiquitinating enzyme USP15 is essential to rescue the E3 ligase Rbx1. Curr Biol.

[B109] Peth A, Berndt C, Henke W, Dubiel W (2007). Downregulation of COP9 signalosome subunits differentially affects CSN complex and target protein stability. BMC Biochem.

[B110] Bornstein G, Ganoth D, Hershko A (2006). Regulation of neddylation and deneddylation of cullin1 in SCFSkp2 ubiquitin ligase by F-box protein and substrate. Proc Natl Acad Sci USA.

[B111] Chew EH, Hagen T (2007). Substrate-mediated regulation of cullin neddylation. J Biol Chem.

[B112] Liu J, Furukawa M, Matsumoto T, Xiong Y (2002). NEDD8 modification of CUL1 dissociates p120(CAND1), an inhibitor of CUL1-SKP1 binding and SCF ligases. Mol Cell.

[B113] Zheng J, Yang X, Harrell JM, Ryzhikov S, Shim EH, Lykke-Andersen K, Wei N, Sun H, Kobayashi R, Zhang H (2002). CAND1 binds to unneddylated CUL1 and regulates the formation of SCF ubiquitin E3 ligase complex. Mol Cell.

[B114] Min KW, Hwang JW, Lee JS, Park Y, Tamura TA, Yoon JB (2003). TIP120A associates with cullins and modulates ubiquitin ligase activity. J Biol Chem.

[B115] Oshikawa K, Matsumoto M, Yada M, Kamura T, Hatakeyama S, Nakayama KI (2003). Preferential interaction of TIP120A with Cul1 that is not modified by NEDD8 and not associated with Skp1. Biochem Biophys Res Commun.

[B116] Goldenberg SJ, Cascio TC, Shumway SD, Garbutt KC, Liu J, Xiong Y, Zheng N (2004). Structure of the Cand1-Cul1-Roc1 complex reveals regulatory mechanisms for the assembly of the multisubunit cullin-dependent ubiquitin ligases. Cell.

[B117] Starostina NG, Lim JM, Schvarzstein M, Wells L, Spence AM, Kipreos ET (2007). A CUL-2 ubiquitin ligase containing three FEM proteins degrades TRA-1 to regulate C. elegans sex determination. Dev Cell.

[B118] Luke-Glaser S, Roy M, Larsen B, Le Bihan T, Metalnikov P, Tyers M, Peter M, Pintard L (2007). CIF-1, a shared subunit of the COP9/signalosome and eukaryotic initiation factor 3 complexes, regulates MEL-26 levels in the Caenorhabditis elegans embryo. Mol Cell Biol.

[B119] Chuang HW, Zhang W, Gray WM (2004). Arabidopsis ETA2, an apparent ortholog of the human cullin-interacting protein CAND1, is required for auxin responses mediated by the SCF(TIR1) ubiquitin ligase. Plant Cell.

[B120] Feng S, Shen Y, Sullivan JA, Rubio V, Xiong Y, Sun TP, Deng XW (2004). Arabidopsis CAND1, an unmodified CUL1-interacting protein, is involved in multiple developmental pathways controlled by ubiquitin/proteasome-mediated protein Degradation. Plant Cell.

[B121] Cheng Y, Dai X, Zhao Y (2004). AtCAND1, a HEAT-repeat protein that participates in auxin signaling in Arabidopsis. Plant Physiol.

[B122] Hwang JW, Min KW, Tamura TA, Yoon JB (2003). TIP120A associates with unneddylated cullin 1 and regulates its neddylation. FEBS Lett.

[B123] Chen X, Zhang J, Lee J, Lin PS, Ford JM, Zheng N, Zhou P (2006). A kinase-independent function of c-Abl in promoting proteolytic destruction of damaged DNA binding proteins. Mol Cell.

[B124] Min KW, Kwon MJ, Park HS, Park Y, Yoon SK, Yoon JB (2005). CAND1 enhances deneddylation of CUL1 by COP9 signalosome. Biochem Biophys Res Commun.

[B125] Menon S, Tsuge T, Dohmae N, Takio K, Wei N (2008). Association of SAP130/SF3b-3 with Cullin-RING ubiquitin ligase complexes and its regulation by the COP9 signalosome. BMC Biochem.

[B126] Das BK, Xia L, Palandjian L, Gozani O, Chyung Y, Reed R (1999). Characterization of a protein complex containing spliceosomal proteins SAPs 49, 130, 145, and 155. Mol Cell Biol.

[B127] Martinez E, Palhan VB, Tjernberg A, Lymar ES, Gamper AM, Kundu TK, Chait BT, Roeder RG (2001). Human STAGA complex is a chromatin-acetylating transcription coactivator that interacts with pre-mRNA splicing and DNA damage-binding factors in vivo. Mol Cell Biol.

[B128] Brand M, Moggs JG, Oulad-Abdelghani M, Lejeune F, Dilworth FJ, Stevenin J, Almouzni G, Tora L (2001). UV-damaged DNA-binding protein in the TFTC complex links DNA damage recognition to nucleosome acetylation. Embo J.

[B129] Liu C, Poitelea M, Watson A, Yoshida SH, Shimoda C, Holmberg C, Nielsen O, Carr AM (2005). Transactivation of Schizosaccharomyces pombe cdt2+ stimulates a Pcu4-Ddb1-CSN ubiquitin ligase. Embo J.

[B130] Higa LA, Banks D, Wu M, Kobayashi R, Sun H, Zhang H (2006). L2DTL/CDT2 interacts with the CUL4/DDB1 complex and PCNA and regulates CDT1 proteolysis in response to DNA damage. Cell Cycle.

